# Mobile Application for Scoliosis Screening Using a Standard 2D Digital Camera

**DOI:** 10.7759/cureus.13944

**Published:** 2021-03-17

**Authors:** Tsutomu Akazawa, Yoshiaki Torii, Jun Ueno, Asako Saito, Hisateru Niki

**Affiliations:** 1 Department of Orthopaedic Surgery, St. Marianna University School of Medicine, Kawasaki, JPN

**Keywords:** scoliosis screening

## Abstract

Several scoliosis detection systems, using three-dimensional (3D) cameras or sensors, have been developed in recent years. Because these systems require specific 3D digital cameras or sensors, and the equipment is expensive, they are rarely used in many countries and regions. The development of a scoliosis screening system that uses standard two-dimensional (2D) digital cameras that come with tablet personal computers (PCs) and smartphones will facilitate the efforts made to detect scoliosis patients on a global scale. The aim of this technical note was to report on a mobile application for scoliosis screening that uses a standard 2D digital camera. The subjects were patients aged 10 years or older who visited our outpatient clinic for scoliosis or suspected scoliosis and underwent whole-spine radiography. Photographs of subjects were obtained using a standard 2D digital camera connected to a tablet PC. For analysis, we used the simplified scoliosis diagnosis support application (Cobb First, Its Corporation, Kawasaki, Japan) which operates on Windows 10 operating system (OS). When an image was imported into the application, it was displayed within a grid. The grid consisted of four columns and 40 rows and was divided into 160 areas. Each image was converted into binarized image data by demarcating skin and background color. The image of the subject was displayed as a black subject on a white background. Two types of conditions were presented to process differences in the environment versus skin color. A binarized image with a clear outline was selected. The determination was displayed as a percentage of the black area in each grid. In each row of the grid, the left and right sides of the black area were compared, and the part with the larger area with respect to the opposite side was colored and displayed. Depending on the ratio of the difference, it was possible to display green, yellow, and red. If this mobile application is available for clinical use, it has the potential to improve the accuracy of screening by physicians and nurses. Furthermore, it may also be used globally to check for possible evidence of scoliosis at home to facilitate the early detection of patients who require a medical checkup for scoliosis. Although it is essential to perform a radiographic examination for the definitive diagnosis of scoliosis, our future goal is to limit radiation exposure and replace a radiologic method with one based on a tablet PC or smartphone. A mobile application using a standard 2D digital camera may improve the accuracy of screening scoliosis by physicians and may have global application in home environments.

## Introduction

Scoliosis screening has been enforced as part of healthcare in schools because of the importance of early detection of scoliosis in teenagers. However, school scoliosis screening is contingent upon subjective assessments via examinations performed by physicians or nurses.

Adam's forward bend test, using a scoliometer, is widely used for scoliosis screening [1]. Nonetheless, it is associated with limitations; it correlates poorly with the Cobb angle, variability across evaluators performing the forward bend test, and the need to manually measure the data within a limited amount of time [2, 3]. Several local governments provide increased funding which permits the use of an objective assessment such as Moiré topography for scoliosis screening [4]. Although Moiré topography is a useful method, it requires special equipment, inspection technicians, and operational costs including equipment maintenance. Moreover, screening using Moiré topography has already been discontinued. Because of cost concerns, a comprehensive school scoliosis screening program has been discontinued in several regions. Furthermore, current scoliosis screening systems have large regional gaps and there are some issues in terms of accuracy [5]. There is a need for a reliable and uniform worldwide screening system.

Several scoliosis detection systems, using three-dimensional (3D) cameras or sensors, have been developed in recent years [6-8]. Because these systems require specific 3D digital cameras or sensors, and the equipment is expensive, they are rarely used in many countries and regions. The development of a scoliosis screening system that uses standard two-dimensional (2D) digital cameras that come with tablet personal computers (PCs) and smartphones will facilitate the efforts made to detect scoliosis patients on a global scale.

The aim of this technical note was to report on a mobile application for scoliosis screening that uses a standard 2D digital camera.

## Technical report

Outline

**Figure 1:**
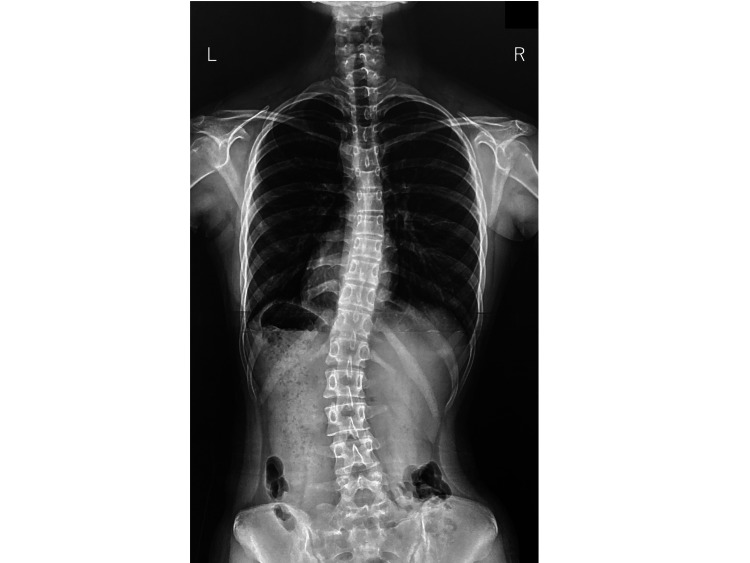
Whole spine radiography. A whole spine radiography was obtained frontally with the patient upright. The radiography showed a 16-degree thoracic curve and a 25-degree lumbar curve.

The subjects were patients aged 10 years or older who visited our outpatient clinic for scoliosis or suspected scoliosis and underwent whole spine radiography (Figure 1). Written informed consent to participate in the research was obtained from the study subjects and/or their parents. Photographs of subjects were obtained using a standard 2D digital camera connected to a tablet PC. For analysis, we used the simplified scoliosis diagnosis support application (Cobb First, Its Corporation, Kawasaki, Japan) which operates on Windows 10 OS. 

Image acquisition

In an isolated booth, physicians photographed the unclothed back of each patient. The patients could choose between a male or a female physician when it came to image acquisition. 

The equipment used in this research was the out-camera (8.0-megapixel digital camera) of a consumer tablet PC (Surface Pro 7, Intel Core i5 processor/8 GB memory/256 GB solid-state drive, OS; Windows 10 Home, Microsoft Corporation, WA). Images taken with a digital camera or smartphone could also be used by importing them into a tablet PC as JPEG images.

**Figure 2:**
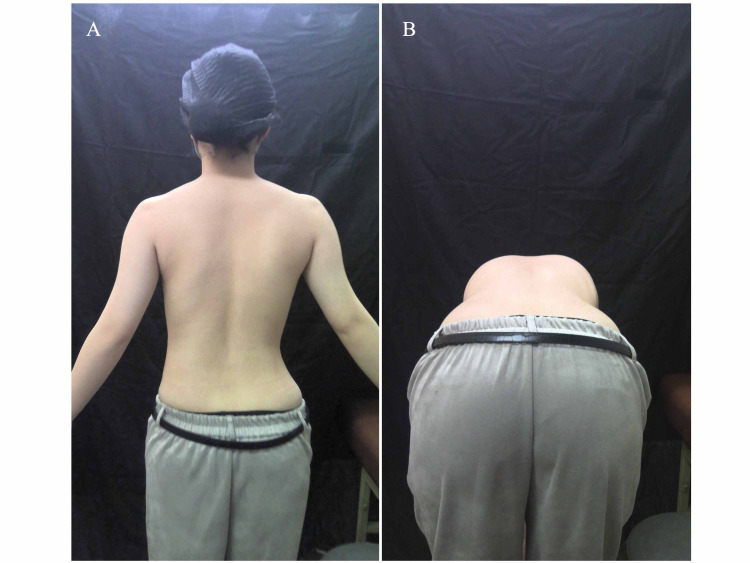
Photographs of the subject. (A) The back of the standing position was photographed. Both upper limbs should have shoulders abduction of about 30 degrees so that the sidelines of the trunk can be seen. (B) The back of the forward bending position was photographed. The patient opened both lower limbs to shoulder width, put both palms together, and took a forward bending position. The patient stopped when the rib or lumbar hump appeared and the image was taken from the back.

The preferred background color was blue or black to enhance the perceptual contrast with patients’ skin color. Flash photography was not used as it caused the skin to glow excessively. First, the posterior view of the standing posture was photographed. The distance between the camera and the subject was not fixed; although the area from the neck to the intergluteal cleft was photographed. Both upper limbs were positioned so that the shoulders were abducted to approximately 30 degrees in order to provide an unobstructed lateral view of the trunk. Whenever hair obscured the neck or shoulders, it was swept up and tied back. Next, a posterior view of the forward bend position was photographed. The patient’s feet were positioned shoulder-width apart, both palms were brought together, and a forward bend position was adopted. When the rib or lumbar hump was exposed, the patient had reached the end position at which an image was taken from the rear (Figure 2).

Image processing

Step 1: Image Capture

**Figure 3:**
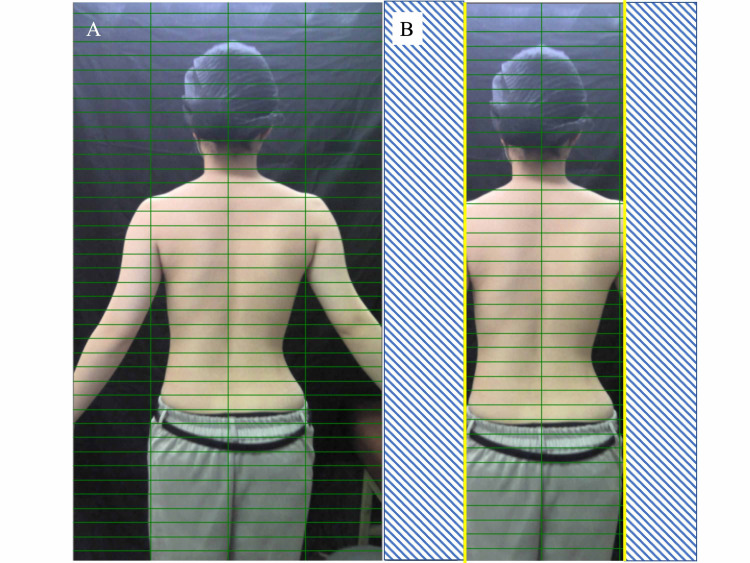
Setting the judgment boundary line. (A) When an image was imported into the application, it was displayed within a grid. The grid consisted of four columns and 40 rows and was divided into 160 areas. (B) The image position was adjusted to the centerline of the grid and set up to coincide with the centerline of the body connecting the C7 spinous process and the intergluteal cleft. The left and right boundary lines were set to fit the axilla. The area outside this boundary was excluded from the analysis.

When an image was imported into the application, it was displayed within a grid. The grid consisted of four columns and 40 rows and was divided into 160 areas (Figure 3A).

Step 2: Setting the Judgment Boundary Line

The background fill color was set by clicking on the background of the image. The image position was adjusted to the centerline of the grid and set up to coincide with the centerline of the body connecting the C7 spinous process and the intergluteal cleft. The left and right boundary lines were set to fit the axilla. The area outside this boundary was excluded from the analysis (Figure 3B). 

Step 3: Setting the Skin Color of the Subject

**Figure 4:**
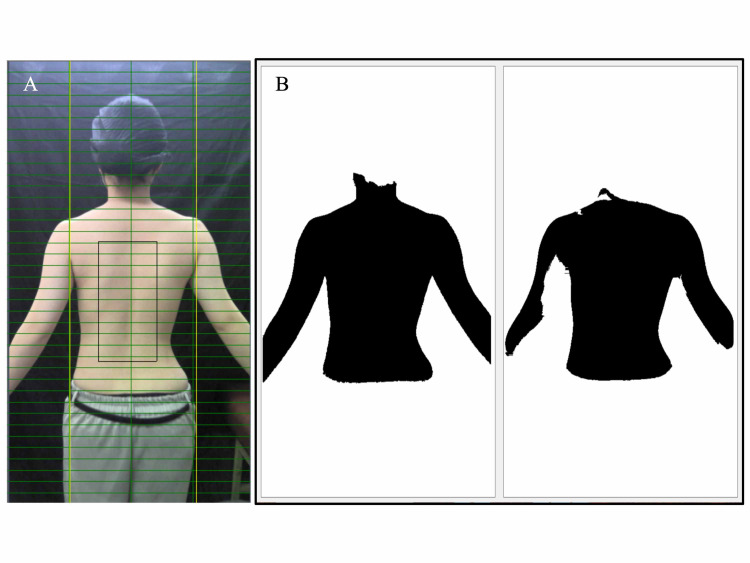
Binarization. (A) A section of the subject’s skin within the range of a square of arbitrary size was chosen as a representative sample of their skin color. (B) Each image was converted into binarized image data by demarcating skin and background color. The image of the subject was displayed as a black subject on a white background. Two types of conditions were presented to process differences in the environment versus skin color.

After assigning the subject a number, a section of the subject’s skin within the range of a square of arbitrary size was chosen as a representative sample of their skin color (Figure 4A).

Step 4: Binarization

Each image was converted into binarized image data by demarcating skin and background color. The image of the subject was displayed as a black subject on a white background. Two types of conditions were presented to process differences in the environment versus skin color (Figure 4B). A binarized image with a clear outline was selected.

Step 5: Judgment

**Figure 5:**
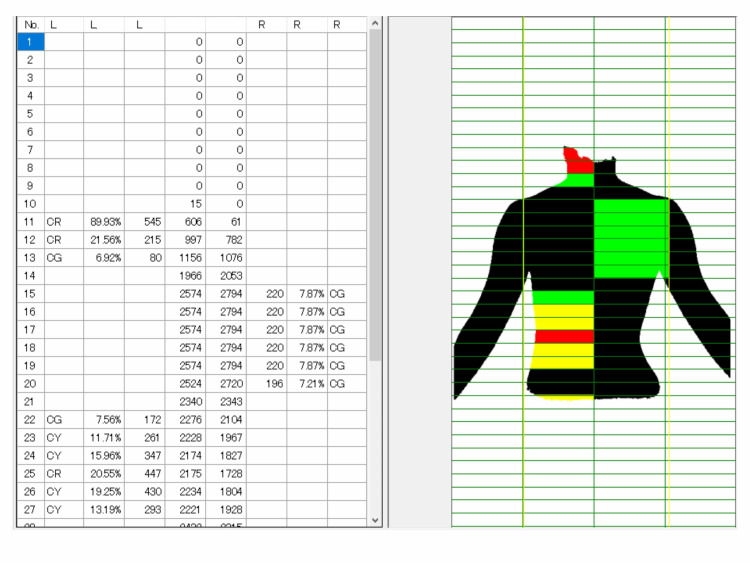
Judgment. In each row of the grid, the left and right sides of the black area were compared, and the part with the larger area with respect to the opposite side was colored and displayed. Depending on the ratio of the difference, it was possible to display green, yellow, and red.

**Figure 6:**
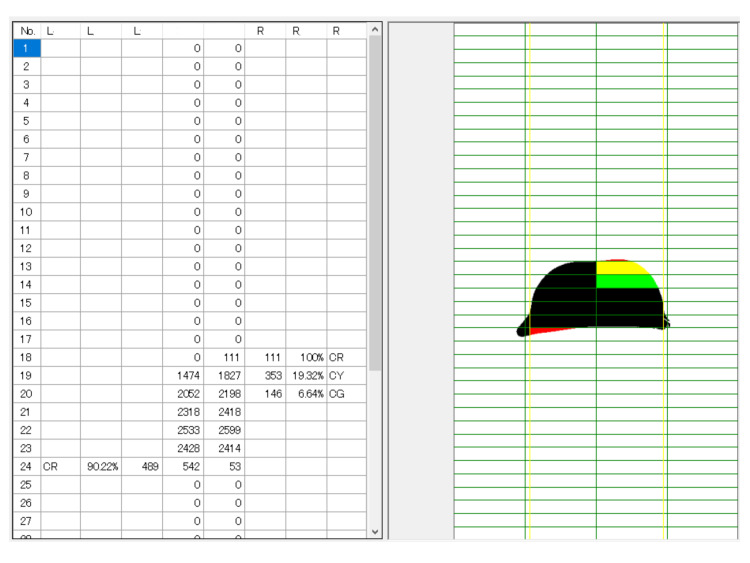
Judgment of the back of the forward bending position. This figure was the result of judging Figure 2B. The right rib hump was detected.

The determination was displayed as a percentage of the black area in each grid. In each row of the grid, the left and right sides of the black area were compared, and the part with the larger area with respect to the opposite side was colored and displayed. Depending on the ratio of the difference, it was possible to display green, yellow, and red (Figures 5 and 6). Data for the judgment results could be saved as a table in an Excel format file. By default, a ratio of five or more and less than 10% was green, 10% or more and less than 20% was yellow, and 20% or more was red. These data were compared with radiographs. The result of the presented case was well accorded with the radiographic data (16-degree thoracic curve and 25-degree lumbar curve).

## Discussion

The advantages provided by our scoliosis screening system are as follows: (i) objective assessments, (ii) a durable application irrespective of equipment changes, (iii) no special operator expertise required, (iv) no need for special devices, (v) less expensive than Moiré topography with its 3D cameras or sensors, and (vi) a uniform screening system. If this mobile application is available for clinical use, it has the potential to improve the accuracy of screening by physicians and nurses. Furthermore, it may also be used globally to check for possible evidence of scoliosis at home to facilitate the early detection of patients who require a medical checkup for scoliosis.

In terms of future work, it will be necessary to determine the appropriate cut-off value for binarized data. For this purpose, it is necessary to establish a larger patient database. The accumulation of a large amount of data will facilitate the diagnosis of scoliosis and prediction of the Cobb angle from 2D images. It has been reported that artificial intelligence (AI) can process large amounts of data in the study of Moiré images of scoliosis [9]. AI can be used in the future as the basis of a system that can be applied to evidence-based treatment that includes curve progression.

## Conclusions

Although it is essential to perform a radiographic examination for the definitive diagnosis of scoliosis, our future goal is to limit radiation exposure and replace a radiologic method with one based on a tablet PC or smartphone. A mobile application using a standard 2D digital camera may improve the accuracy of screening scoliosis by physicians and may have global application in home environments.
